# The Influence of Bacteria-Inoculated Mineral Fertilizer on the Productivity and Profitability of Spring Barley Cultivation

**DOI:** 10.3390/plants12061227

**Published:** 2023-03-08

**Authors:** Justinas Anušauskas, Dainius Steponavičius, Kęstutis Romaneckas, Kristina Lekavičienė, Ernestas Zaleckas, Eglė Sendžikienė

**Affiliations:** 1 Faculty of Engineering, Agriculture Academy, Vytautas Magnus University, Studentu Str. 15A, LT-53362 Akademija, Kaunas District, Lithuania; 2 Faculty of Agronomy, Agriculture Academy, Vytautas Magnus University, Studentu Str. 11, LT-53361 Akademija, Kaunas District, Lithuania; 3 Faculty of Forest Sciences and Ecology, Agriculture Academy, Vytautas Magnus University, Studentu Str. 11, LT-53361 Akademija, Kaunas District, Lithuania

**Keywords:** bacterial inoculant, biologically enriched, mineral fertilization, barley yield, income, cultivation efficiency

## Abstract

The heavy use of mineral fertilizers causes imbalances in the biological processes that take place in soil. Therefore, it is necessary to develop more effective fertilizers or fertilizer complexes that ensure agricultural productivity and soil conservation. There is currently a lack of knowledge regarding the effectiveness of biologically enriched, complex mineral fertilizers for spring barley fertilization. The hypothesis of this study was that bacteria-enriched (*Paenibacillus azotofixans*, *Bacillus megaterium*, *Bacillus mucilaginosus*, and *Bacillus mycoides*), complex mineral fertilizers (N_5_P_20.5_K_36_) have significant impacts on the yield and potential for economic use of spring barley. Experimental studies were carried out for three years (2020–2022) with sandy loam soil in southern Lithuania. Four different spring barley fertilization scenarios (SCs) were investigated. In SC-1 (control), complex mineral fertilizer (N_5_P_20.5_K_36_) was not applied. In the other SCs, spring barley was sown with a drill and fertilizers were incorporated locally during the sowing operation: fertilization scenario SC-2 used 300 kg ha^−1^, SC-3 used 150 kg ha^−1^ preceded by a bacteria-inoculated complex mineral fertilizer (N_5_P_20.5_K_36_), and SC-4 used 300 kg ha^−1^ with the same bacterial complex. The results showed that the bacterial inoculant increased the efficiency of the mineral fertilizer and had an effect on plant growth in barley. For three consecutive years in the same plots, the bacterial inoculant showed significant positive effects on grain yield (changes of 8.1% in 2020, 6.8% in 2021, and 17.3% in 2022 between SC-2 and SC-4). Comparing the several different fertilizer scenarios from an economic point of view, it was observed that the highest profit per hectare was obtained with SC-4 in all three years of the study. Comparing SC-4 and SC-2, an increase of 13.7% was observed in 2020, followed by 9.1% and 41.9% in 2021 and in 2022, respectively. This study will be useful for farmers, biological inoculant manufacturers, and scientists researching the effectiveness of biological inoculants for growing agricultural crops. We found that it is possible to increase the yield of barley (7–17%) using the same rate of mineral fertilization by enriching it with bacterial inoculants. Further studies should be conducted to determine the effects of the bacterial inoculant on crop yield and soil over a period longer than 3 years.

## 1. Introduction

The depletion of natural resources, energy consumption, and environmental problems associated with fertilizer production processes and fertilizer use are driving a shift towards more sustainable resources [1,2]. Intensive tillage and the heavy use of mineral fertilizers and chemical plant protection products cause imbalances in the biological processes that take place in soil, leading to declines in soil quality [3,4]. To address these problems, various technologies can be applied; these technologies must not negatively impact human health but still be biologically and economically effective [5]. Organic farming aims to reduce the environmental impact of agricultural production by avoiding the use of synthetic chemical compounds, including fertilizers and pesticides, and promoting practices such as crop diversification [6] and the use of organic fertilizers [7]. 

Biofertilizers are currently receiving attention as a better alternative to chemical fertilizers in terms of protecting the environment and human health. Biofertilizers contain various living microorganisms, including bacteria and cyanobacteria, which cover the rhizospheres or inner spaces of plants when they are sprinkled on seeds, the plant surface, and/or the soil. These microorganisms promote plant growth by converting essential nutrients (mainly nitrogen, phosphorus, and potassium) from non-absorbable to absorbable forms [8,9,10]. Several years of research by Gavelienė et al. [11] has shown that microbial biostimulants can have a positive influence not only on yield but also on quality by increasing the amount of nutrients and bioactive compounds present that are beneficial to human health.

Biswas et al. [12] recommended the use of mineral fertilizers in combination with biological inoculants. In their paper, they indicated that mineral fertilizers with lower efficiency—in their case, low-grade rock phosphates (LGRPs)—should be inoculated with phosphate-solubilizing bacteria (PSB). In their studies, a PSB-inoculated LGRP program increased intrinsic microbial activity by 10–24%, reduced the P fixation capacity by altering the P adsorption chemistry, and optimized the P supply parameter. The authors indicated that the use of PSB and LGRP could reduce the use of standard phosphate fertilizer by 50% without adversely affecting yield.

Potassium (K) is a macronutrient that has a major impact on plant metabolism, growth rate, and development [13]. However, plants can only utilize 2–10% of the total K present in the soil. Most K (90%) is present as a stable compound in silicate minerals, such as feldspar and mica, which are difficult for plants to take up and utilize [14]. For this reason, potassium-solubilizing bacteria (KSB) have been used as a biofertilizer in place of mineral fertilizers to enrich soil and improve its quality [15,16]. This inoculant can release the K present in stable soil minerals through chelation, complexolysis, exchange reactions, acidolysis, and organic acid production. It also releases other minerals present in the soil, such as Si, Fe, and Ca [14,17]. Han (2006) found that the KSB inoculant, when applied to soil, had a positive effect on the uptake of potassium in a variety of crops, including cotton, cucumbers, and oilseed rape [18]. 

Vejan et al. [19] argued that controlled-release fertilizers (CRFs) play a crucial and necessary role in the sustainable agricultural industry. CRFs not only reduce nutrient dissipation due to evaporation and leaching but also provide physiological and biochemical support for plant growth. Li et al. [20] reported that the agricultural fertilizer industry and researchers have been focusing on the comprehensive use of bio-based materials. The application of nanotechnology can contribute significantly to improvements in sustainable agriculture through syntheses of slow- or controlled-release fertilizers in various ways. CRFs—both with and without nanotechnology—are available as advanced and intelligent fertilizer systems [21]. 

In summary, the addition of suitably selected microorganisms or other biologically active components to mineral fertilizers can both contribute to the conversion of chemicals to a form acceptable to plants and provide a transport function, ensuring that the chemical elements required by the plants are taken up by the roots and do not pollute the environment. However, the literature lacks studies on the effectiveness of the use of mineral fertilizers enriched with a bacterial complex for the fertilization of spring barley.

In this work, we aimed to determine the effect of bacteria-inoculated, complex mineral fertilizer on the biometric parameters and yield of spring barley while also evaluating the cost-effectiveness of the fertilizer.

## 2. Results and Discussion

### 2.1. Spring Barley Productivity Indicators

**Figure 1:**
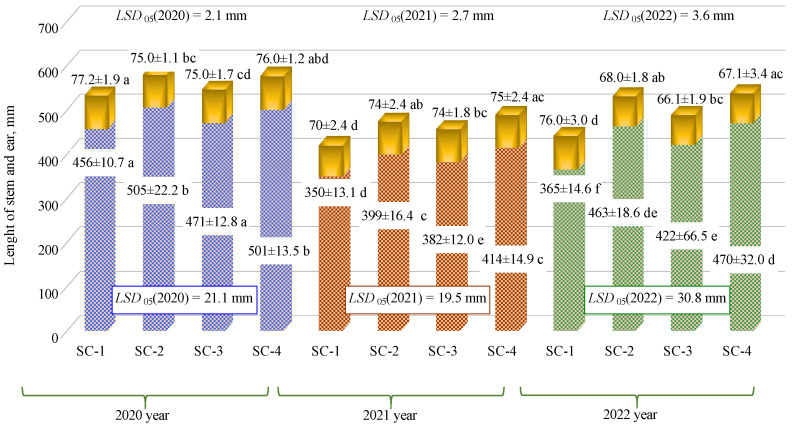
Effects of different fertilization strategies on barley stem and ear lengths. Matching letters indicate no significant difference between scenarios in the same years. A *t*-test was used for statistical analysis.

The three-year experimental study (2020, 2021, and 2022) showed that, in all years, barley stem lengths were higher in SC-2, SC-3, and SC-4 than in SC-1 (control) (Figure 1). When comparing SC-1 with SC-2, SC-3, and SC-4, the greatest differences in barley stem lengths (26.85%, 15.62%, and 28.77%, respectively) were found in the third year of the study, while the smallest differences (10.74%, 3.29%, and 9.87%, respectively) were found in the first year of the study. This was because, in both spring and autumn in the third year, the soil potassium content was higher (2.94% in autumn and 11.88% in spring) in SC-4 than in SC-1. However, in the first year, no increase in soil potassium was detected in SC-4. In all years of the study, the greatest difference in stem lengths was observed in SC-4, when 300 kg ha^−1^ of biologically enriched mineral fertilizer was applied. Although potassium and phosphorus were not higher in SC-4 soil in any of the years of the study when compared to the other scenarios, the bacterial inoculant increased the availability of these nutrients to the plants. Previous research has suggested that potassium- and phosphate-solubilizing inoculants containing mycorrhizal fungi may improve the uptake of potassium and phosphorus in plants by increasing their diffusion rate and significantly increasing the fixation of minerals [22,23,24]. The microorganisms in bacterial inoculants change root functions (root exudation) [25], improve the carbohydrate metabolism and nutrient and water uptake of the cultivated plant [26], and influence the rhizosphere population [27]. Other researchers have found that the use of products containing phosphorus-dissolving microorganisms increased the height of cannabis plants [28]. Another study showed that the use of potassium-dissolving bacteria significantly increased (23.53%) the height of garlic, which was influenced by increased potassium content of 28.41 mg kg^−1^ in the soil [29]. In 2020, no significant difference was found when comparing SC-1 with SC-3 with the lowest application rate (150 kg ha^−1^) for the biologically enriched, complex mineral fertilizer, while in the other years, a significant difference was found when comparing SC-1 with SC-3. For the control groups in 2021 and 2022, substantial differences were found in all cases when compared to the other three scenarios (SC-2, SC-3, and SC-4). The same trend was observed in all years of the study, with no significant differences in barley stem lengths between SC-2 (complex fertilizer rate: 300 kg ha^−1^) and SC-4 (complex fertilizer rate: 300 kg ha^−1^, biologically enriched). 

The experimental trials showed that, in 2021, ear length was significantly lower in SC-1—by about 4.33 mm on average—when compared to SC-2, SC-3, and SC-4 (Figure 1). In the second year of the study, barley ear lengths varied between 74 mm and 75 mm in SC-2, SC-3, and SC-4. In 2020, the barley ear length was significantly greater in SC-1 than in SC-2 and SC-3; in 2022, it was significantly greater than that in SC-2, SC-3, and SC-4. When comparing data between years, greater ear and stem lengths were observed in 2020 than in 2021 and 2022. This was due to the better meteorological conditions in the first year of the study. 

**Figure 2:**
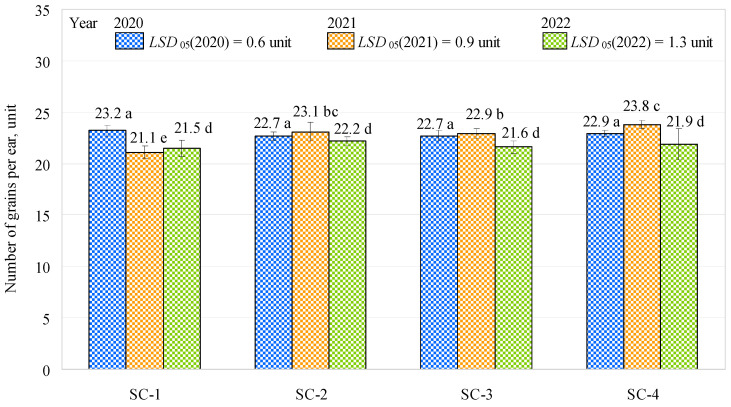
Effects of different fertilization strategies on the numbers of grains per ear. Matching letters indicate no significant difference between scenarios in the same years. Error bars represent the 95% confidence interval of the mean. A *t*-test was used for statistical analysis.

Although the length of the barley ears was greater in the first year of the study than the second and third years, the numbers of grains per ear in the first year of the study did not differ significantly and were similar (about 22.9 grains on average) between all the treatments (SC-1, SC-2, SC-3, and SC-4), with 23.2, 22.7, 22.7, and 22.9 grains, respectively (Figure 2). In the second year of the study, the numbers of grains per ear significantly increased by 9.47% (SC-2), 8.53% (SC-3), and 12.80% (SC-4) when compared to the control (SC-1). However, increased potassium (approximately 4.5%) and humus (approximately 3%) were observed only in SC-4 compared to SC-1. pH was most optimal in SC-1 and SC-2. No significant differences were observed when comparing SC-2 with SC-3 or SC-2 with SC-4; however, there was a significant difference between SC-3 and SC-4, with the number of grains per ear being about 4% higher in SC-4 (complex fertilizer rate: 300 kg ha^−1^, biologically enriched) than in SC-3 (complex fertilizer rate: 150 kg ha^−1^, biologically enriched). In the third year of the study, no significant differences were found between the variants, and the average number of grains per ear was about 21.8 units. In terms of the numbers of grains per ear, the second year of the study showed an improvement in the effectiveness of the bacterial inoculant compared to the first and third years of the study. This may have been influenced by the higher humus content in the soil in the spring in the second year of the study, which varied from 2.00% (SC-2) to 2.21% (SC-4). Humus absorbs water and retains it in the soil, which was particularly beneficial in the dry second year (2021) of the survey. The study by Khan et al. [22] confirms these findings, as the authors stated that the use of bacterial inoculants helped retain moisture in the soil under arid conditions. Marulanda et al. [30] also reported an improvement in plant tolerance to drought stress due to drought-tolerant microorganisms.

**Figure 3:**
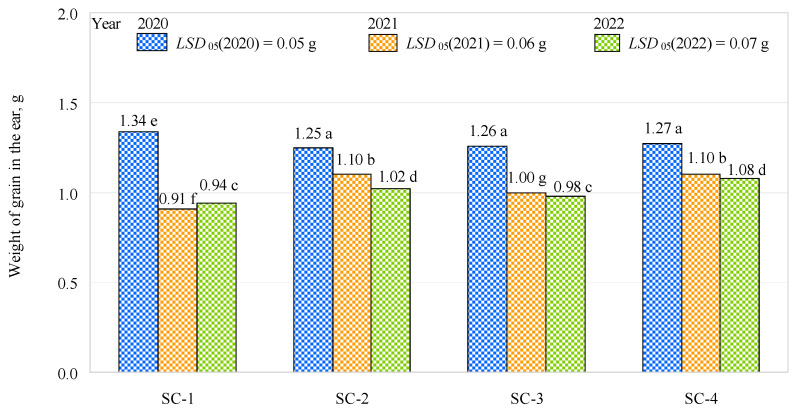
Effects of different fertilization strategies on the weights of grain in the ear. Matching letters indicate no significant difference between scenarios in the same years. A *t*-test was used for statistical analysis.

The experimental trials showed that, in the first year of the study, the weight of the grain in the ear was significantly higher in the control variant—about 0.08 g or 6% on average—when compared to the other variants (Figure 3). This may have been influenced by the higher potassium and humus content in the spring in the first year of the study in SC-1 compared to the other scenarios. Furthermore, the pH of the soil was determined to be optimal (5.5). In addition, in the first year of research, the bacterial inoculant could have had less influence on the increase in grain weight. Comparing SC-2, SC-3, and SC-4 with each other, no significant differences were found between the variants. In the second year of the study, when comparing the control with SC-2, SC-3, and SC-4, the trend was the opposite of that in the first year of the study. The control scenario showed a significantly lower weight for the grain in the ear than SC-2, SC-3, and SC-4 (17%, 9%, and 17%, respectively). Grain weight in the ear was also significantly lower (0.1 g or 9%) in SC-3 than in SC-2 or SC-4. In the third year of the study, no significant differences were observed in the weights of the grain in the ear when comparing the control with SC-3, which had a complex fertilizer rate of 150 kg ha^−1^ (biologically enriched), and when comparing SC-2 (complex fertilizer rate of 300 kg ha^−1^) with SC-4 (complex fertilizer rate of 300 kg ha^−1^, biologically enriched). Significantly higher weights were found in SC-2 and SC-4 compared to the control (about 8% and about 15%, respectively); in addition, the weights in SC-2 and SC-4 were about 4% and 10% higher than in SC-3. Comparing the three years of the study, the effect of the bacterial inoculant on the weight of grain in the ear was greater in the second year than in the first and third years. This was due to the higher number of grains per ear in the second year of the trial than in the first and third years of the trial. In the second year of the survey, a greater proportion of humus was found in the soil compared to the other years. The bacterial inoculant, therefore, led to more efficient uptake of water by the plant roots. 

**Figure 4:**
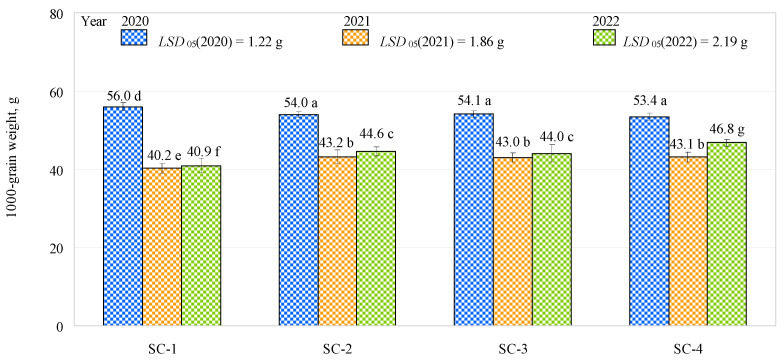
Effects of different fertilization strategies on 1000 grain weight. Matching letters indicate no significant difference between scenarios in the same years. Error bars represent the 95% confidence interval of the mean. A *t*-test was used for statistical analysis.

The 1000 grain weight varied from 53.4 g (SC-4) to 56 g (control) in the first year of the study, from 40.2 g (control) to 43.2 g (SC-2) in the second year, and from 40.93 g (control) to 46.7 g (SC-4) in the third year (Figure 4). In the first and second years of the study, the 1000 grain weights were similar (53.83 g and 43.1 g on average, respectively) for SC-2, SC-3, and SC-4, but they were significantly lower (about 4%) in 2020 and significantly higher (about 7%) in 2021 for SC-1. In the third year of the study, when comparing the control with the other three scenarios studied, the 1000 grain weight was significantly lower in the control. In the third year of research, the amount of phosphorus in the soil in SC-1 was found to be higher than in the other scenarios, the amount of potassium in the soil was found to be higher only in SC-4 compared to SC-1, and the amount of humus was observed to be higher in autumn in all scenarios compared to SC-1. The bacterial inoculant showed an advantage in the third year of the study, with increased absorption of nutrients and water by the plants due to root exudation, resulting in a greater 1000 grain weight. Despite the fact that soil contains high levels of phosphorus, it is poorly available to plants due to its extremely low diffusion rate and significant fixation by soil minerals; therefore, phosphorus-solubilizing microorganisms can be used to improve its uptake by plants [23,24,31,32,33,34]. The greatest 1000 grain weight (46.80 g) was obtained in SC-4 and was significantly higher than that in SC-2 and SC-3 (by about 5% and 6%, respectively). No significant differences were found when comparing SC-2 with SC-3. In the third year of the study, the efficiency of the bacterial inoculant was improved, as the 1000 grain weight was significantly higher in SC-4 (complex fertilizer rate: 300 kg ha^−1^, biologically enriched) than in SC-1 (control), SC-2 (complex fertilizer rate: 300 kg ha^−1^), and SC-3 (complex fertilizer rate: 150 kg ha^−1^, biologically enriched). This could have been influenced by SC-4 having the highest soil potassium and humus content compared to the other scenarios. However, the soil pH in SC-4 was approximately 13% lower than the optimum. These findings show that it is possible to improve results without increasing the fertilizer rate by using bacterial inoculants. Phosphate-solubilizing microorganisms in bacterial inoculants have also been reported to improve phosphorus availability to plants [22,34]. This is important because phosphorus is an essential macronutrient during plant growth and development, but it is a very immobile soil nutrient and poorly available to plants due to its extremely low diffusion speed [3,31,32]. 

**Figure 5:**
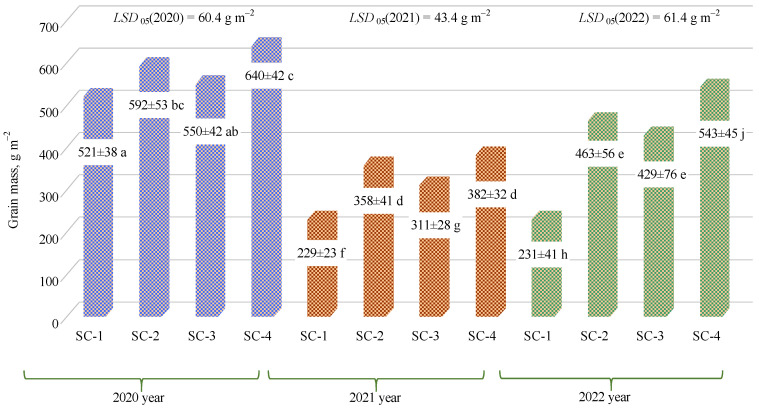
Effects of different fertilization strategies on grain weight. Matching letters indicate no significant difference between scenarios in the same years. Error bars represent the 95% confidence interval of the mean. A *t*-test was used for statistical analysis.

In the first year of the study, the grain weight varied from 521 g m^−2^ to 640 g m^−2^; in the second year, from 229 g m^−2^ to 382 g m^−2^; and in the third year, from 231 g m^−2^ to 543 g m^−2^. In all years, grain weight was highest in SC-4 and lowest in SC-1 (Figure 5). When evaluating the three years of research, the use of the biological reparation only significantly increased grain weight in the third year in SC-4 (complex mineral fertilizer rate of 300 kg ha^−1^, biologically enriched) compared to SC-2 (complex mineral fertilizer rate of 300 kg ha^−1^, not biologically enriched). One study reported that mineral fertilizer with bacterial impregnation performed better than mineral fertilizer without impregnation as a result of the enhanced availability of nutrients and increased wheat yield by 20% [35]. Xiao et al. [36] reported that microbial inoculations significantly increased rice yield by up to 17.73%.

The number of barley ears varied from 699.1 units m^−2^ (SC-1) to 875.2 units m^−2^ (SC-4) in 2020, from 507.6 units m^−2^ (SC-1) to 750.1 units m^−2^ (SC-4) in 2021, and from 453.3 units m^−2^ (SC-1) to 798.8 units m^−2^ (SC-4) in 2022 (Table 1). For all years, the fact that the highest numbers of barley ears were observed in SC-4 could have been a result of the high potassium content detected in the soil in autumn (2020: 87 mg kg^−1^, 2021: 108 mg kg^−1^, 2022: 101 mg kg^−1^) compared to the other studied years. In the third year of the study, the differences in the numbers of ears in SC-2, SC-3, and SC-4 compared to the control were greater than in the first and second years of the study. This was influenced by the reduced potassium, phosphorus, and humus contents in the soil in SC-1 in the third year. In the first year, the number of ears was 142.1 units m^−2^ higher in SC-2 than in the control, 69.4 units m^−2^ higher in SC-3 than in the control, and 176.1 units m^−2^ higher in SC-4 than in the control. In the second year, the number of ears was 179.7 units m^−2^ higher in SC-2 than in the control, 118.3 units m^−2^ higher in SC-3 than in the control, and 242.5 units m^−2^ higher in SC-4 than in the control. In the third year of the study, the number of ears was 275.9 units m^−2^ higher in SC-2 than in the control, 198.8 units m^−2^ higher in SC-3 than in the control, and 345.5 units m^−2^ higher in SC-4 than in the control. In 2021 and 2022, substantial differences in the numbers of barley ears per square meter were found between all the variants studied. In 2021 and 2022, the number of barley ears per square meter was found to be significantly lower in SC-3 compared to SC-2 and SC-4; however, in both years, the number of barley ears per square meter was found to be significantly higher in SC-4 (complex fertilizer rate of 300 kg ha^−1^, biologically enriched) than in SC-2 (complex fertilizer rate of 300 kg ha^−1^), which was influenced by the effect of the bacterial inoculants. The amount of potassium and humus in the soil increased in SC-4 (compared to SC-2) with the help of the bacterial inoculant, although the pH was slightly lower (about 13% on average) than the optimum (5.5). The use of bacterial inoculants containing phosphate-solubilizing microorganisms not only improved crop yields but also reduced dependence on limited resources [37].

The barley ear-to-grain ratio was higher in 2021 than in 2020 and 2022 in all scenarios studied (Table 1). In the first year of the study, the ear-to-grain ratio varied from 1.35 units g^−1^ (SC-1) to 1.43 units g^−1^ (SC-2); in the second year, from 2 units g^−1^ (SC-4) to 2.27 units g^−1^ (SC-1); and in the third year, from 1.5 units g^−1^ (SC-4) to 2.01 units g^−1^ (SC-1). Significant differences were observed in the first year of the study when comparing SC-1 with SC-2, in the second year of the study when comparing SC-1 with SC-3 and SC-4, and in the third year of the study when comparing SC-1 with all the other scenarios studied, as well as when comparing SC-3 with SC-4. 

**Figure 6:**
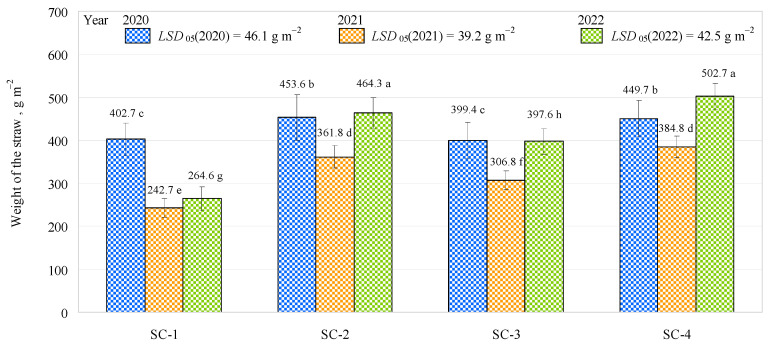
Effects of different fertilization strategies on the weight of straw. Matching letters indicate no significant difference between scenarios in the same years. Error bars represent the 95% confidence interval of the mean. A *t*-test was used for statistical analysis.

The experimental trials showed that the weight of the straw varied from 399.4 g m^−2^ (SC-3) to 453.6 g m^−2^ (SC-2) in 2020, from 242.7 g m^−2^ (SC-1) to 384.8 g m^−2^ (SC-4) in 2021, and from 264.6 g m^−2^ (SC-1) to 502.7 g m^−2^ (SC-4) in 2022 (Figure 6). Ahmad et al. [35] reported that the bacterial impregnation of mineral fertilizers increased wheat straw yield from 19.6 to 24.2 g per pot. Comparing SC-1 with the other scenarios investigated in the first year of the study, the weight of the straw in SC-1 was found to be significantly lower than in SC-2 and SC-4 (by about 11% and 10%, respectively), while no significant difference was found in comparison to SC-3. In addition, no significant difference was found between SC-2 and SC-4. In the second year of the study, the weight of the straw was found to be significantly lower in SC-1 than in SC-2, SC-3, and SC-4 by 33%, 21%, and 37%, respectively. No significant differences were found between SC-2 and SC-4, and the weight of the straw was similar (about 373.3 g m^−2^). In the third year of the study, no significant differences in the weight of the straw were found between SC-2 and SC-4, which showed an average weight of about 483.5 g m^−2^. However, when comparing SC-1 with the other scenarios studied, the weight of the straw in SC-1 was significantly lower. This was due to the lower stem lengths in SC-1.

Summarizing the results of the experimental trials, it can be concluded that bacterial inoculants have a positive impact on barley productivity. Barley productivity was improved in SC-4 (complex fertilizer rate of 300 kg ha^−1^, biologically enriched) compared to SC-1 (control), SC-2 (complex fertilizer rate of 300 kg ha^−1^), and SC-3 (complex fertilizer rate of 150 kg ha^−1^, biologically enriched). It is particularly important to note that, without increasing the fertilizer rate (300 kg ha^−1^), we achieved better results with biologically enriched fertilizer than with the application of mineral fertilizer. Moreover, taken together, the results of the three-year study suggest that a long-term study would show an advantage in SC-3 over SC-2. Sankar Biswas et al. [12] suggested that the use of commercial phosphorus fertilizers could be decreased by 50% without compromising the yield of wheat crops and recommended the use of phosphate-solubilizing bacteria inculcated with low-grade rock phosphates. Other researchers have argued that new tools to optimize microbiological processes are needed to improve soil fertility [38]. This is relevant because there are also concerns about the decline in global phosphorus supplies associated with reaching peak phosphorus mining reserves [39,40].

### 2.2. Results of Economic Evaluation

**Figure 7:**
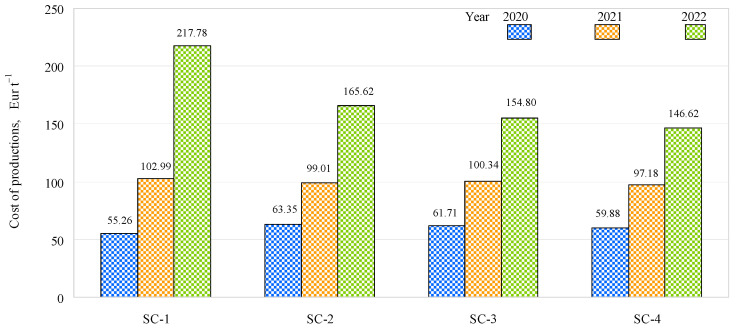
Effects of different fertilization strategies on the cost of production.

The cost of production was the lowest in 2020 and the highest in 2022 in all scenarios studied, mainly due to significant increases in economic indicators (Figure 7). In 2020, the lowest cost of production was EUR 55.26 t^−1^ in SC-1, which was lower than that in SC-2, SC-3, and SC-4 by EUR 8.09 t^−1^, EUR 6.45 t^−1^, and EUR 4.62 t^−1^, respectively. In 2021, the cost of production was slightly higher in SC-1 compared to SC-2, SC-3, and SC-4: by 3.98 EUR t^−1^, 2.65 EUR t^−1^, and 5.81 EUR t^−1^, respectively. In 2022, the cost of production was significantly higher in SC-1 than in the other scenarios studied, with increases of EUR 52.16 t^−1^, EUR 62.98 t^−1^, and EUR 71.16 t^−1^ compared to SC-2, SC-3, and SC-4, respectively. In 2022, a significant difference in the cost of production was found for SC-1 when compared to SC-2, SC-3, and SC-4, mainly due to the degradation of the soil during the three years of the study caused by the fertilization scenarios without complex fertilizer. 

**Figure 8:**
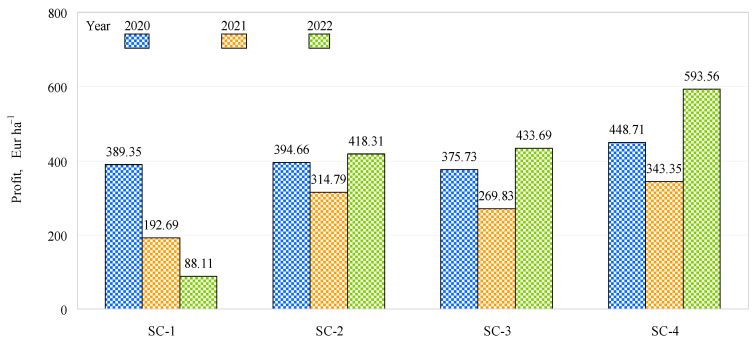
Effects of different fertilization strategies on profit.

In 2020, the highest profit (448.7 EUR ha^−1^) was obtained in SC-4, demonstrating a higher figure than SC-1 (389.4 EUR ha^−1^), SC-2 (394.7 EUR ha^−1^), and SC-3 (375.7 EUR ha^−1^) (Figure 8). In 2021, SC-4 again had the highest profit (EUR 343.4 ha^−1^), EUR 150.7 ha^−1^, EUR 28.6 ha^−1^, and EUR 73.6 ha^−1^ higher than in SC-1, SC-2, and SC-3, respectively. In 2022, the profit varied from 88.1 EUR ha^−1^ (SC-1) to EUR 593.6 ha^−1^ (SC-4) and was significantly higher in SC-4 than in SC-1, SC-2, and SC-3. Comparing the other scenarios studied in both 2020 and 2021 and comparing SC-4 between different years, the highest profit was obtained in 2022 with SC-4. 

SC-4 had the lowest costs in 2021 and 2022; in addition, the profit was the highest in this scenario, as the interaction between the bacterial inoculants and the fertilizer had the greatest positive effect on barley productivity. Microbial inoculation is a promising method for improving crop yields in an economically and environmentally friendly way [36]. In agreement with our results, Naujokienė et al. [41] argued that inoculating soil with biological preparations can reduce costs and increase profits. 

## 3. Materials and Methods

### 3.1. Site Description

The experimental field trials were carried out from 2020 to 2022 in the Alytus district, Lithuania, on a farm owned by the farmer M. Anušauskas. The region is characterized by Endoeutric Albeluvisol (*Orthieutric Albeluvisol*). With respect to soil texture, the research field was classified as sandy loam soil. A field with low fertility and low phosphorus and potassium contents was chosen for the study so that the effect of the bacterial inoculant would be more significant.

### 3.2. Experimental Design and Agronomic Practice

**Figure 9:**
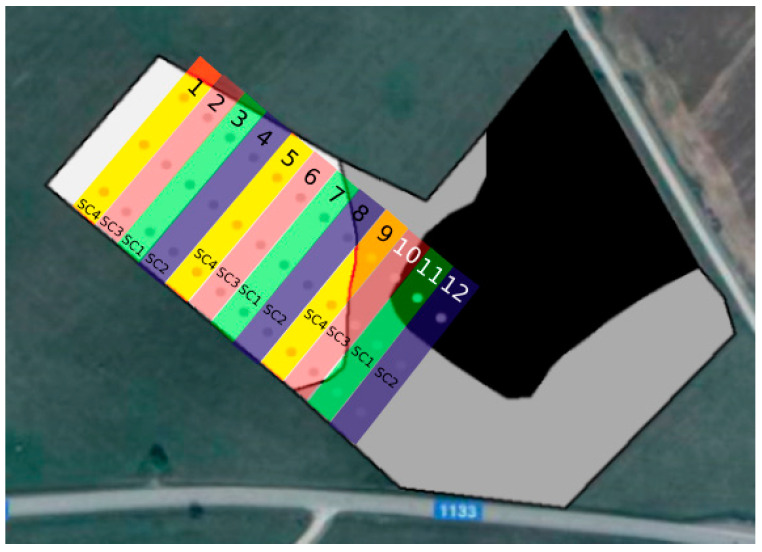
Arrangement of experimental plots.

The trials were carried out in the same field on 2 ha of land (longitude X 24.212825; latitude Y 54.481939) for three consecutive years. Four fertilizer technologies were used in the study; thus, the field was divided into 12 parts (each 12 m wide and 75 m long) and the experiment was carried out with three repetitions. The layout of the fields was systemic (Figure 9). 

The first fertilization scenario (SC-1; control), which did not include P or K fertilization, was assigned to plots 3, 7, and 11. In the following fertilization scenarios, complex mineral fertilizer (N_5_P_20.5_K_36_) was applied at different rates. The mineral fertilizer N_5_P_20.5_K_36_ consists of 5% total nitrogen (N) (ammonium nitrogen), 20.5% total P_2_O_5_ (20.5% water-soluble P_2_O_5_), and 36% water-soluble K_2_O. This fertilizer mixture consisted of ammonium phosphate (NH_4_H_2_PO_4_) and potassium chloride (KCl). NH_4_H_2_PO_4_ is a fertilizer that contains up to 12.0% N and 61.7% phosphorus (P_2_O_5_). KCl is a fertilizer containing 53.0–61.9% K_2_O; it is moderately hygroscopic and dissolves well in water [42]. In the second fertilization scenario (SC-2), 300 kg ha^−1^ was applied in plots 4, 8, and 12. The plots for the third scenario (SC-3; plots 2, 6, and 10) were fertilized with 150 kg ha^−1^ (N_5_P_20.5_K_36_) enriched (coated) with a bacterial complex (*Paenibacillus azotofixans*, *Bacillus megaterium*, *Bacillus mucilaginosus*, and *Bacillus mycoides* in equal concentrations totaling 1 × 10^9^ cfu g^−1^) at a rate of 500 g ha^−1^, which equaled 0.33% of the total mass of one granule. The fourth fertilization scenario (SC-4; plots 1, 5, and 9) used the same rate of bacterial inoculant, equaling 0.17% of the total mass of one granule, with 300 kg ha^−1^ of complex mineral fertilizer (N_5_P_20.5_K_36_). Nitrogen fertilization was applied uniformly over the whole field at the end of tillering (BBCH 25–30) with ammonium nitrate (NH_4_NO_3_; N_34.4_) at a rate of 68.8 kg N ha^−1^ (Table 2). The amount of nitrogen (N_total_) differed in the different scenarios due to the unequal application rates for the complex mineral fertilizer. Therefore, the yield difference between scenarios SC-2 and SC-4, which used identical amounts of mineral fertilizers, was highlighted in the Results and Discussion section.

The trials were carried out with *Hordeum vulgare* L. (cv. Iron) spring barley. The tillage technology used for all four scenarios did not differ, with plowing in the autumn and seed bed preparation in the spring before sowing. During sowing, a 4 m working width suspended disc drill was used and a seed placement of about 30–40 mm, 142 mm row spacing, and a seed rate of 4.0–4.5 million units per hectare. Complex mineral fertilizer (N_5_P_20.5_K_36_) was applied at sowing. Nitrogen fertilization was carried out using a two-disc fertilizer spreader. 

In the spring of 2020, the electrical conductivity of the soil was determined before sowing using a Veris 3150 MSP soil testing machine (USA). After processing the data using https://fieldfusion.veristech.com/ (accessed on 10 April 2020), an apparent soil electrical conductivity map was produced (Figure S1).

After evaluating the electrical conductivity zones for the soil, it was found that the texture of the field soil selected for research did not significantly differ and was within the range of 2.30–7.17 mS m^−1^. In addition, no soil samples or barley harvest samples were taken from the zone with the highest (4.86–7.17 mS m^−1^) apparent electrical conductivity (which was about 5% of the research field). As a result, the unevenness of the soil did not distort the results of the research.

Soil samples were taken from each test plot (1–12, Figure 9) from at least 15 locations and the soil was mixed. Using precise coordinates (from an EZ-GUIDE 250 GPS device), samples were taken at the same locations every spring and autumn for three consecutive years.

The soil samples were analyzed at the Agrochemical Research Laboratory (Kaunas, Lithuania). They were subjected to agrochemical analyses to determine the amount (mg kg^−1^) of available phosphorus and potassium using the Egner–Riehm–Domingo (A–L) method (LVP D-07:2016), the humus content (%) using the Tyurin method, and the soil pH_KCl_ using the potentiometric method (determined in 1 M KCl) (ISO 10390:2005—soil quality and determination of pH). After obtaining the results from the individual plots, averages were derived.

Background soil samples were obtained in April 2020 before the research began. It was determined that the soil pH was 5.3–5.6 (slightly acidic), the P_2_O_5_ concentration was 66–88 mg kg^−1^ (low phosphorus), the K_2_O concentration was 77–96 mg kg^−1^ (low potassium), and the humus concentration was 1.50–1.78% (low) (Table 3). From the humus concentration, the total nitrogen (N_total_ (%)) in the soil could be calculated using the formula determined by Adamaviečiene et al. [43]:

Y=0.269+7.286·x, %

where Y is the amount of humus (%) and x is N_total_ (%). The total amount of nitrogen in the soil was calculated as 0.17–0.21.

### 3.3. Plant Sampling

Plant sampling was carried out by taking 10 random samples from each of the 12 plots (Figure 8), resulting in a total of 30 samples per scenario and 120 samples for all trials. It should be mentioned again that the samples were not taken from the 4.86–7.17 mS m^−1^ soil electrical conductivity zone. The samples were taken with a 50 × 50 cm frame placed on the soil and all barley plants that fell within the hoop were cut. The cut samples were numbered and placed in bags, then transported to the laboratory for further analysis.

### 3.4. Determination of Plant Yield and Biometric Parameters

The plant samples were analyzed in the laboratory of the Vytautas Magnus University Agriculture Academy. For each sample, 30 representative plants were taken. The stem length (mm) and ear length (mm) were measured; the grains were husked, counted (units), and weighed (g); and the remaining straw was weighed (g). All the remaining ears in the sample (units) were also counted. The samples were then threshed using a stationary *Wintersteiger* LD 350 laboratory threshing bench (Wintersteiger GmbH, Austria). The threshed grains and the rest of the straw were then weighed (g). The masses of all barley grains present in the sample were summed (g), and the masses of all the straw in the sample were also summed (g). In addition, the number of barley ears (unit) in the sample was added up. These results were converted into the number of ears, the weight of grain, and the weight of the straw per square meter of test plot (m^2^). In the following calculations, the weight of the grain was recalculated for 14% moisture, while the weight of straw was recalculated for 18% moisture. With these data, the ear-to-grain ratio was calculated. All 30 samples from each fertilization scenario were analyzed and the average of the yield and the biometric parameters were derived.

### 3.5. Methodology of Economic Evaluation 

To assess the economic benefits associated with the use of bacterial inoculants, it was necessary to consider the total costs of the fertilizer scenarios in terms of barley production, the yields obtained, and the grain prices on the market at that time (Tables S1–S3).

When evaluating agricultural cultivation and processing technologies, profit gain (*G*) per unit area (hectare) is usually calculated as follows:

$$G=\left(P-C\right)\cdot Y,\;\mathrm{EUR}\;{\mathrm{ha}}^{-1}$$

where *P* is the grain market price (EUR t^−1^), *C* is the cost of the harvest (EUR t^−1^), and *Y* is the grain yield (t ha^−1^).

The yield (*Y*) from the obtained sample results in g m^−2^ was converted to t ha^−1^.

The prime cost of the harvest was calculated as the ratio between the expenses of the production technology (EUR ha^−1^) and the yield (t ha^−1^):

$$C=E\cdot Y^{-1},\;\mathrm{EUR}\;t^{-1}$$

where *E* represents the expenses of the production technology (EUR ha^−1^).

### 3.6. Meteorological Conditions

Meteorological conditions were recorded between the months of April and August from 2020 to 2022 at a Lithuanian hydrometeorological service station. It was located about 12 km away from the trial field (Figures S2–S4). 

Total precipitation between April and August in 2020 was 350.9 mm, with an average air temperature of 14.5 °C. The month of April was exceptionally dry, with only 4 mm of precipitation. By 31 June, total precipitation equaled 51.4 mm. Between April and August 2021, the average temperature rose to 15.2 °C and total precipitation was 366 mm. The months of May and August stood out, with 122 mm of precipitation monthly and temperatures of 19.5 °C and 16.4 °C, respectively. It should also be noted that the average air temperature in July reached 22.5 °C, higher than the average air temperatures of 17.3 °C and 18.0 °C in 2020 and 2022, respectively. Between April and August 2022, the average air temperature was 14.8 °C and total precipitation was 339 mm. July had the highest precipitation and was warmer than the two preceding years (38.7 mm and 20.9 °C compared to 92.8 mm and 18.6 °C in 2020 and 122 mm and 16.4 °C in 2021).

### 3.7. Statistical Analysis

Data points are presented as mean values with their confidence levels (at a probability *p* ≤ 0.05), and the least significant difference *LSD*_05_ was calculated using a *t*-test at a probability *p* ≤ 0.05 with Statistica 10.0 statistical software (TIBCO Software, Inc., Palo Alto, CA, USA).

The arithmetic mean of each studied parameter (e.g., grain weight (g m^−2^), plant numbers (units m^−2^), lengths of barley stems and ears (mm), etc.) was obtained after 30 repetitions. Differences between the means of the tested variants were judged to be significant when they were equal to or greater than the calculated least significant difference limit *LSD*_05_ [44]:

$$LSD_{05}=t_{table}\sqrt{MS_{e}\left(\frac{1}{n_{a}}+\frac{1}{n_{b}}\right)},$$

where *MS_e_* is the variance error between the data means or the dispersion acquired from ANOVA tables; *t_table_* is Student’s criteria value taken from the tables in accordance with the number of degrees of freedom and the probability level (*p* ≤ 0.05 in this study); and *n_a_* and *n_b_* are the numbers of replications for the two variants.

## 4. Conclusions

Our three-year study showed a positive effect from the application of bacterial inoculant on spring barley yield. The results showed an increase in barley yield without a corresponding increase in the fertilizer rate when the fertilizer was biologically enriched. The application of the bacterial inoculant significantly increased (6.81–17.25%) the barley grain yield in SC-4 (complex fertilizer rate: 300 kg ha^−1^, biologically enriched) compared to SC-2 (complex fertilizer rate: 300 kg ha^−1^). This was likely due to the fact that the bacterial inoculants used converted insoluble phosphorus and potassium compounds into their soluble forms, which are more readily available to plants. The highest efficiency for the biological inoculant was found in the third year of the study. This was due to increases in the proportions of phosphorus (18.7%) and potassium (13.0%) in the soil in the third year of the trial compared to the first year.

The bacterial inoculant also had a positive impact on economic indicators. In terms of the cost of production and profit, the third year of the study showed the highest efficiency. In the third year, the lowest cost of production (146.62 EUR t^−1^) and the highest profit (593.56 EUR ha^−1^) were obtained in SC-4 (complex fertilizer rate: 300 kg ha^−1^, biologically enriched) when compared to SC-1, SC-2, and SC-3. A 41.89% increase in profit was found in SC-4 compared to SC-2 (complex fertilizer rate: 300 kg ha^−1^), and a 36.86% increase was observed when compared to SC-3 (complex fertilizer rate: 150 kg ha^−1^, biologically enriched).

As this study showed that the maximum positive effect of the bacterial inoculant was achieved in the third year, it is recommended that further studies be carried out to determine its effects on cereal yields and soil over a period longer than 3 years. 

It would also be relevant to investigate the effects of site-specific seeding and variable-rate precision fertilization technologies on the yield and quality indicators of spring barley when the fertilizers used are biologically enriched.

## Figures and Tables

**Table 1:** Effects of different fertilization strategies on the numbers of ears and the ear-to-grain ratios of barley.

Indicator	2020				2021				2022			
	SC-1	SC-2	SC-3	SC-4	SC-1	SC-2	SC-3	SC-4	SC-1	SC-2	SC-3	SC-4
Number of barley ears, unit m^−2^	699.1 ± 41.3 ^a^	841.2 ± 70.6 ^bc^	768.5 ± 57.4 ^ab^	875.2 ± 64.2 ^c^	507.6 ± 35.2 ^a^	687.3 ± 47 ^b^	625.9 ± 43.7 ^c^	750.1 ± 48.3 ^d^	453.3 ± 21.5 ^a^	729.2 ± 22.2 ^b^	652.1 ± 21.6 ^c^	798.8 ± 21.9 ^d^
*t*-test	*LSD*_05_ (2020) = 81.3 units m^−2^				*LSD*_05_ (2021) = 60.1 units m^−2^				*LSD*_05_ (2022) = 61.9 units m^−2^			
Barley ear-to-grain ratio, unit g^−1^	1.35 ± 0 ^a^	1.43 ± 0.1 ^b^	1.4 ± 0.1 ^ab^	1.37 ± 0.1 ^ab^	2.27 ± 0.1 ^a^	2.08 ± 0.3 ^ab^	2.05 ± 0.1 ^b^	2 ± 0.1 ^b^	2.01 ± 0.2 ^c^	1.6 ± 0.1 ^ab^	1.7 ± 0.2 ^a^	1.5 ± 0 ^b^
*t*-test	*LSD*_05_ (2020) = 0.069 units g^−1^				*LSD*_05_ (2021) = 0.222 units g^−1^				*LSD*_05_ (2022) = 0.183 units g^−1^			

Note: matching letters indicate no significant difference between scenarios in the same year.

**Table 2:** Fertilization scenarios.

Fertilization Scenario	Dosage of Fertilizer		Total Amount of Macroelement		
	At Sowing Time	At BBCH 25–30	Nitrogen N_tot_, kg ha^−1^	Phosphorus P_tot_, kg ha^−1^	Potassium K_tot_, kg ha^−1^
SC-1 (control)	0.0	200 kg ha^−1^ NH_4_NO_3_ N_34.4_	68.8	0.0	0.0
SC-2	300 kg ha^−1^ N_5_P_20.5_K_36_		83.8	61.5	108.0
SC-3	150 kg ha^−1^ N_5_P_20.5_K_36_ + 0.5 kg ha^−1^ bacteria		76.3	30.8	54.0
SC-4	300 kg ha^−1^ N_5_P_20.5_K_36_ + 0.5 kg ha^−1^ bacteria		83.8	61.5	108.0

**Table 3:** Agrochemical properties of the soil.

Period	pH				P_2_O_5_, mg kg^−1^			
	SC-1	SC-2	SC-3	SC-4	SC-1	SC-2	SC-3	SC-4
Spring 2020	5.5 ± 1.2	5.6 ± 1.8	5.3 ± 1.0	5.3 ± 1.3	88 ± 71	72 ± 60	68 ± 36	66 ± 25
Autumn 2020	5.4 ± 1.2	5.4 ± 1.7	4.8 ± 0.8	4.9 ± 0.5	82 ± 50	90 ± 159	45 ± 26	57 ± 26
Spring 2021	5.6 ± 1.8	5.6 ± 1.6	5.1 ± 1.0	5.0 ± 0.9	75 ± 64	54 ± 48	50 ± 26	53 ± 19
Autumn 2021	5.2 ± 1.0	5.4 ± 1.7	4.9 ± 0.6	4.8 ± 0.8	99 ± 68	60 ± 17	75 ± 33	75 ± 22
Spring 2022	5.2 ± 1.6	5.5 ± 2.1	4.8 ± 0.9	4.8 ± 0.7	83 ± 57	79 ± 60	56 ± 30	74 ± 23
Autumn 2022	5.2 ± 1.6	5.5 ± 2.3	5.4 ± 1.1	4.7 ± 0.2	79 ± 54	76 ± 53	60 ± 26	65 ± 10
	K_2_O, mg kg^−1^				Humus, %			
	SC-1	SC-2	SC-3	SC-4	SC-1	SC-2	SC-3	SC-4
Spring 2020	96 ± 37	87 ± 13	77 ± 11	84 ± 23	1.74 ± 0.22	1.58 ± 0.67	1.78 ± 0.38	1.50 ± 0.31
Autumn 2020	76 ± 21	72 ± 16	70 ± 16	87 ± 11	1.80 ± 0.40	1.95 ± 0.15	1.84 ± 0.63	1.70 ± 0.24
Spring 2021	95 ± 27	81 ± 17	78 ± 7	92 ± 17	2.14 ± 0.58	2.00 ± 0.23	2.04 ± 0.35	2.21 ± 0.21
Autumn 2021	102 ± 15	80 ± 22	89 ± 15	108 ± 11	2.05 ± 0.31	1.83 ± 0.44	1.92 ± 0.61	1.87 ± 0.28
Spring 2022	99 ± 29	86 ± 20	82 ± 18	102 ± 8	1.68 ± 0.35	1.51 ± 0.27	1.52 ± 0.25	1.60 ± 0.33
Autumn 2022	89 ± 43	79 ± 13	71 ± 10	101 ± 10	1.57 ± 0.33	1.69 ± 0.36	2.11 ± 1.06	1.85 ± 0.54

## Data Availability

The data presented in this study are available in the article.

## Supplementary Materials

The following supporting information can be downloaded at: https://www.mdpi.com/article/10.3390/plants12061227/s1, Figure S1: Apparent soil electrical conductivity map (mS m^−1^); Table S1: Economic indicators for 2020 [45]; Table S2: Economic indicators for 2021 [46]; Table S3: Economic indicators for 2022 [47]; Figure S2: Meteorological data for April–August 2020; Figure S3: Meteorological data for April–August 2021; Figure S4: Meteorological data for April–August 2022.
