# Supplementary material for: The Influence of Bacteria-Inoculated Mineral Fertilizer on the Productivity and Profitability of Spring Barley Cultivation
Source: Plants (Basel). 2023 Mar 8;12(6):1227. doi: 10.3390/plants12061227 (PMC10059695; doi:10.3390/plants12061227)
Supplement: Supplementary file 1 [file plants-12-01227-s001.zip › plants-2203091-supplementary.pdf]

plants
MDPI

## Supplementary Materials

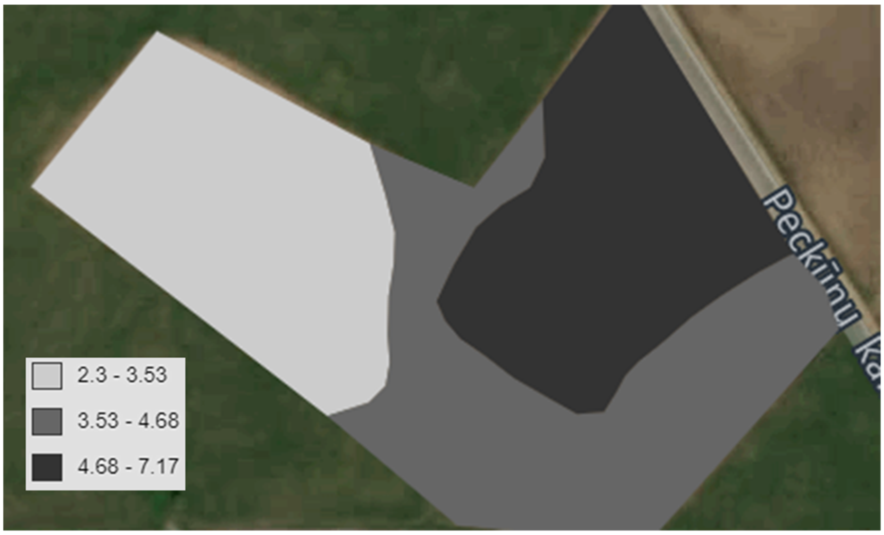


**Figure S1.** Apparent soil electrical conductivity map (mS m$^{-1}$).

**Table S1.** Economic indicators for 2020 (from the financial statements for the farm owned by M. Anušauskas).

| Product | Price, EUR kg$^{-1}$ | Production Technology, kg ha$^{-1}$ | | | | Expenses Related to Production Technology, EUR ha$^{-1}$ | | | |
|---|---|---|---|---|---|---|---|---|---|
| | | SC-1 | SC-2 | SC-3 | SC-4 | SC-1 | SC-2 | SC-3 | SC-4 |
| Seed | 0.42 | 230.00 | 230.00 | 230.00 | 230.00 | 96.60 | 96.60 | 96.60 | 96.60 |
| **NPK** | 0.29 | 0.00 | 300.00 | 150.00 | 300.00 | 0.00 | 87.30 | 43.65 | 87.30 |
| Bacteria | 16.00 | 0.00 | 0.00 | 0.50 | 0.50 | 0.00 | 0.00 | 8.00 | 8.00 |
| Fungicide | 50.42 | 1.00 | 1.00 | 1.00 | 1.00 | 50.42 | 50.42 | 50.42 | 50.42 |
| Growth regulator no. 1 | 7.00 | 0.50 | 0.50 | 0.50 | 0.50 | 3.50 | 3.50 | 3.50 | 3.50 |
| Ammonium nitrate | 0.25 | 200.00 | 200.00 | 200.00 | 200.00 | 50.00 | 50.00 | 50.00 | 50.00 |
| Herbicide | 40.00 | 0.25 | 0.25 | 0.25 | 0.25 | 10.00 | 10.00 | 10.00 | 10.00 |
| Growth regulator no. 2 | 21.36 | 0.50 | 0.50 | 0.50 | 0.50 | 10.68 | 10.68 | 10.68 | 10.68 |
| Insecticide | 26.70 | 0.30 | 0.30 | 0.30 | 0.30 | 8.01 | 8.01 | 8.01 | 8.01 |
| Diesel | 0.69 | 85.00 | 85.00 | 85.00 | 85.00 | 58.65 | 58.65 | 58.65 | 58.65 |
| | | | | | Total | 287.86 | 375.16 | 339.51 | 383.16 |
| | | | | | | Barley grain market price, EUR t$^{-1}$ | | | 130.00 |

**Table S2.** Economic indicators for 2021 (from the financial statements for the farm owned by M. Anušauskas).

| Product | Price, EUR kg$^{-1}$ | Production Technology, kg ha$^{-1}$ | | | | Expenses Related to Production Technology, EUR ha$^{-1}$ | | | |
|---|---|---|---|---|---|---|---|---|---|
| | | SC-1 | SC-2 | SC-3 | SC-4 | SC-1 | SC-2 | SC-3 | SC-4 |
| Seed | 0.20 | 230.00 | 230.00 | 230.00 | 230.00 | 46.00 | 46.00 | 46.00 | 46.00 |
| **NPK** | 0.39 | 0.00 | 300.00 | 150.00 | 300.00 | 0.00 | 117.98 | 58.99 | 117.98 |
| Bacteria | 34.48 | 0.00 | 0.00 | 0.50 | 0.50 | 0.00 | 0.00 | 17.24 | 17.24 |
| Fungicide | 50.42 | 1.00 | 1.00 | 1.00 | 1.00 | 50.42 | 50.42 | 50.42 | 50.42 |

| | | | | | | | | | |
|---|---|---|---|---|---|---|---|---|---|
| Growth regulator no. 1 | 7.00 | 0.50 | 0.50 | 0.50 | 0.50 | 3.50 | 3.50 | 3.50 | 3.50 |
| Ammonium nitrate | 0.30 | 200.00 | 200.00 | 200.00 | 200.00 | 59.29 | 59.29 | 59.29 | 59.29 |
| Herbicide | 22.52 | 0.25 | 0.25 | 0.25 | 0.25 | 5.63 | 5.63 | 5.63 | 5.63 |
| Growth regulator no. 2 | 21.36 | 0.50 | 0.50 | 0.50 | 0.50 | 10.68 | 10.68 | 10.68 | 10.68 |
| Insecticide | 26.70 | 0.30 | 0.30 | 0.30 | 0.30 | 8.01 | 8.01 | 8.01 | 8.01 |
| Diesel | 0.62 | 85.00 | 85.00 | 85.00 | 85.00 | 52.70 | 52.70 | 52.70 | 52.70 |
| | | | | | Total | 236.23 | 354.21 | 312.46 | 371.45 |
| | | | | | | Barley grain market price EUR $t^{-1}$ | | | 187.00 |

**Table S3.** Economic indicators for 2022 (from the financial statements for the farm owned by M. Anušauskas).

| Product | Price, EUR $kg^{-1}$ | Production Technology, kg $ha^{-1}$ | | | | Expenses Related to Production Technology, EUR $ha^{-1}$ | | | |
|---|---|---|---|---|---|---|---|---|---|
| | | SC-1 | SC-2 | SC-3 | SC-4 | SC-1 | SC-2 | SC-3 | SC-4 |
| Seed | 0.22 | 230.00 | 230.00 | 230.00 | 230.00 | 50.60 | 50.60 | 50.60 | 50.60 |
| **NPK** | 0.88 | 0.00 | 300.00 | 150.00 | 300.00 | 0.00 | 264.60 | 132.30 | 264.60 |
| Bacteria | 58.08 | 0.00 | 0.00 | 0.50 | 0.50 | 0.00 | 0.00 | 29.04 | 29.04 |
| Fungicide | 50.42 | 1.00 | 1.00 | 1.00 | 1.00 | 50.42 | 50.42 | 50.42 | 50.42 |
| Growth regulator no. 1 | 4.24 | 0.50 | 0.50 | 0.50 | 0.50 | 2.12 | 2.12 | 2.12 | 2.12 |
| Ammonium nitrate | 1.34 | 200.00 | 200.00 | 200.00 | 200.00 | 267.40 | 267.40 | 267.40 | 267.40 |
| Herbicide | 37.20 | 0.25 | 0.25 | 0.25 | 0.25 | 9.30 | 9.30 | 9.30 | 9.30 |
| Growth regulator no. 2 | 15.80 | 0.50 | 0.50 | 0.50 | 0.50 | 7.90 | 7.90 | 7.90 | 7.90 |
| Insecticide | 26.70 | 0.30 | 0.30 | 0.30 | 0.30 | 8.01 | 8.01 | 8.01 | 8.01 |
| Diesel | 1.25 | 85.00 | 85.00 | 85.00 | 85.00 | 106.25 | 106.25 | 106.25 | 106.25 |
| | | | | | Total | 502.00 | 766.60 | 663.34 | 795.64 |
| | | | | | | Barley grain market price EUR $t^{-1}$ | | | 256.00 |

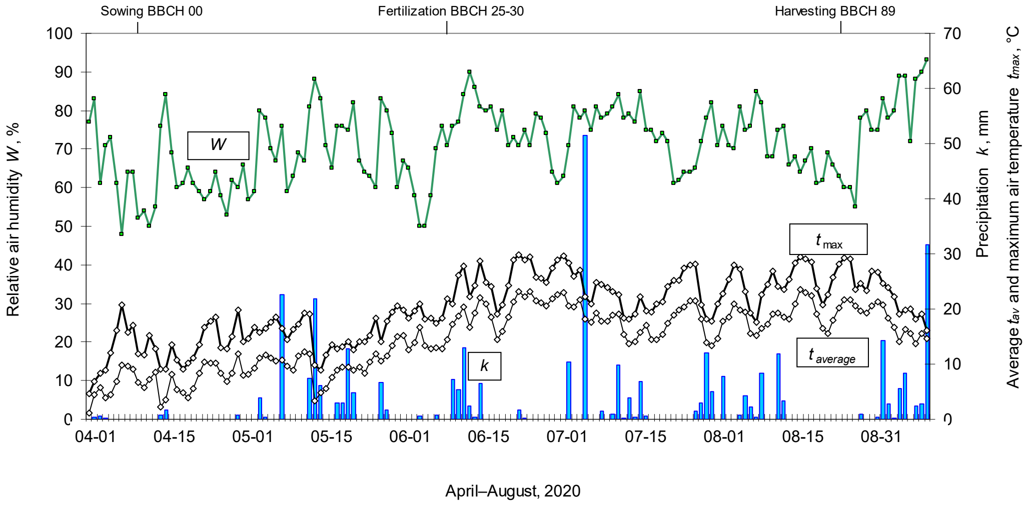


**Figure S2.** Meteorological data for April–August 2020.

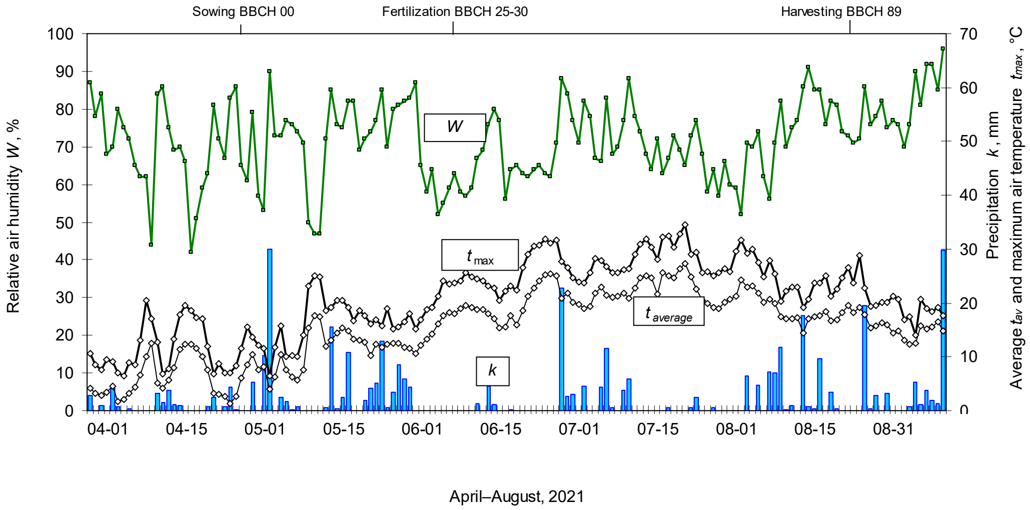


**Figure S3.** Meteorological data for April–August 2021.

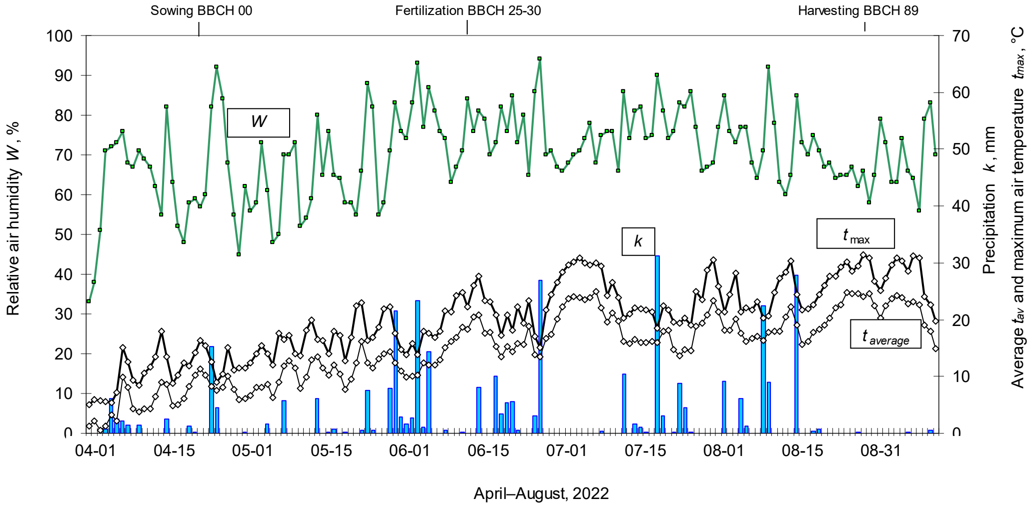


**Figure S4.** Meteorological data for April–August 2022.
